# The Influence of Traffic Noise on Appreciation of the Living Quality of a Neighborhood

**DOI:** 10.3390/ijerph8030777

**Published:** 2011-03-07

**Authors:** Dick Botteldooren, Luc Dekoninck, Dominique Gillis

**Affiliations:** 1 Department of Information Technology, Ghent University, St. Pietersnieuwstraat 41, 9000 Gent, Belgium; E-Mail: luc.dekoninck@intec.ugent.be; 2 Department of Mobility and Spatial Planning, Ghent University, Vrijdagmarkt 10/301, 9000 Gent, Belgium; E-Mail: dominique.gillis@ugent.be

**Keywords:** traffic noise, quality of life, neighborhood satisfaction

## Abstract

Traffic influences the quality of life in a neighborhood in many different ways. Today, in many patsy of the world the benefits of accessibility are taken for granted and traffic is perceived as having a negative impact on satisfaction with the neighborhood. Negative health effects are observed in a number of studies and these stimulate the negative feelings in the exposed population. The noise produced by traffic is one of the most important contributors to the appreciation of the quality of life. Thus, it is useful to define a number of indicators that allow monitoring the current impact of noise on the quality of life and predicting the effect of future developments. This work investigates and compares a set of indicators related to exposure at home and exposure during trips around the house. The latter require detailed modeling of the population’s trip behavior. The validity of the indicators is checked by their ability to predict the outcome of a social survey and by outlining potential causal paths between them and the outcome variables considered: general satisfaction with the quality of life in the neighborhood, noise annoyance at home, and reported traffic density in the area.

## 1. Introduction

In societies where basic needs are largely fulfilled, attention to mental well-being is growing. The quality of the living environment is one of the determinants for this well-being [1]. Therefore there is also a growing interest in how the quality of a neighborhood is to be assessed and preserved. The appreciation of the living quality of a neighborhood depends on various indicators, which can be grouped into personal attributes, attributes of the house and the characteristics of the neighborhood [2]. Early studies have examined the living density of the neighborhood as a determinant for quality of life [3–5]. In [2] it is shown that the type of the area where the house is located is more useful in predicting individual neighborhood satisfaction than variables relating to the individual respondent like age, sex or economic status. The type of accomodation (detached house, semi-detached, flat) also contributed significantly to this model. Other references, however, prove the impact of several resident types (‘advantaged’, ‘generally satisfied’, ‘settled’, ‘withdrawn’, ‘indifferent’, ‘insecure’), each reporting different reasons to (dis)like their neighbourhoods [6].

Traffic has a significant impact on the quality of a living environment. Traffic grants access to neighborhood functions and the rest of the world, but this positive aspect is largely taken for granted in many parts in the world. Negative impacts include safety, air pollution, smell and noise, but it is mainly noise annoyance that is perceived as a burden and threat to the everyday life quality of the neighboorhood. Noise annoyance is also determined by attributes of the person, the house and the neighborhood. The most stable personal factor is ‘subjective noise sensitivity’ [7,8] which is an important predictor of noise annoyance [9,10]. In [10] it is shown that other significant indicators include person-related variables (age, years of employment, Paykel stress score, duration of stay at the accomodation during the day), house-related variables (windows of living room and/or bedroom oriented toward street, floor) and neighborhood-related variables (noise levels as equivalent noise level L_eq_ for the daytime and nighttime periods, the maximal nighttime noise level L_max_, , traffic flow during day and during the night).

This paper presents part of an ongoing quest for models that allow assessment of the impact of land use and mobility planning on the quality of the living environment. It attempts to quantify the relationship between street traffic, traffic noise as an important impact of traffic, traffic noise annoyance as an important effect of traffic noise, and the quality of life in a neighborhood. A classical methodology based on a questionnaire survey on the one hand and exposure indicators on the other, is followed. The uniqueness of the paper lies in the introduction of additional exposure indicators for noise exposure away from the dwelling. This exposure is related to trips that people living in a certain neighborhood might make and the traffic modus they are likely to use. Special care is taken to accurately aggregate the exposure both within one trip and between trips. It will be shown that the best available indicator for noise exposure on the road significantly improves models for noise annoyance based on a more classical equivalent noise level on the façade L_Aeq_. It will also be shown that this indicator very significantly outperforms façade exposure when it comes to modeling reported quality of life in the neighborhood. It even models perceived traffic intensity better than the number of vehicles passing in front of the dwelling.

**Figure 1.**
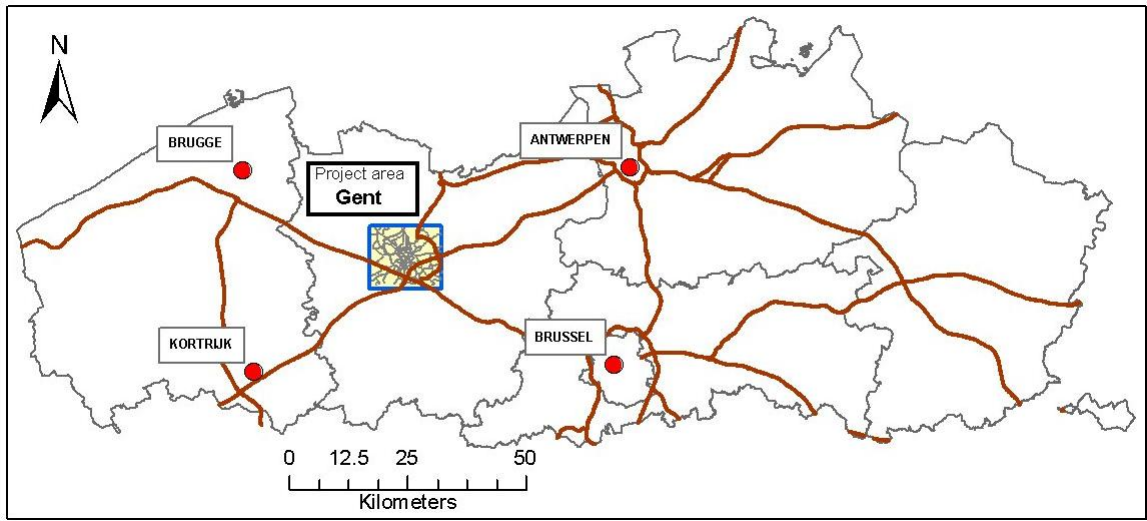
Regional positioning of the project area (blue) within the Flanders, showing the highways (brown) and the major surrounding cities (red).

The proposed indicators for traffic noise and pathways to the appreciation of the quality of the living environment were validated and tested on Flanders, the northern part of Belgium and—for the more detailed modeling—on Gent, a 350,000 inhabitant city in the center of the region. This context may affect some of the details of the conclusions so it is discussed in somewhat more detail. Flanders has about 6 million inhabitants living on 13,500 square kilometers. About one quarter of this area is a built-up area. The largest city in the region is Antwerp, with about 470,000 inhabitants. Thus mega-city problems are not expected in the study area. Transport is attracted to several sea harbors: Antwerp, Gent, Zeebrugge, Oostende, together handling about 250 million tons of goods/year. In addition Flanders is very close to the Dutch harbors of Rotterdam and Duinkerken. Flanders is also surrounded by several big cities: Brussels, the capital of Belgium, Paris, Amsterdam, … influencing strongly the amount of traffic on the major arteries. The location of the project area is represented in Figure 1.

The city of Gent has a historical center that is now mainly a recreational and shopping area that is largely free of car and truck traffic, but has an extended tram and bus system. The surrounding areas include a ring road connecting the harbor to the main highways E17 and E40. The E17 connects Antwerp and The Netherlands to France, the E40 connects Brussels and parts of Germany to the coast, France and the channel tunnel to England. Both highways pass within 4 km of the city center. Thus the selected study area is heavily loaded with traffic (mainly goods transport) external to the area and thus well suited to investigate the influence of traffic on the appreciation of the living environment.

## 2. Method

### 2.1. Survey

This study relies on an existing series of surveys conducted every three years by the Department for Environment, Nature, and Energy of the Flemish Government for subjective evaluation. This written survey (Schriftelijk Leefomgevings Onderzoek or SLO) addresses the quality of the living environment in general and annoyance caused by noise, odor, and light in particular. Three years of surveys are considered: SLO0 conducted in 2001 with 3,200 participants, SLO1 conducted in 2004 with 5,000 participants and SLO2 conducted in 2008 again with 5,000 participants. The response rates of the written survey were relatively high (65%, 63%, and 56%, respectively) due to a telephone recruitment of participants prior to sending out the survey. Due to this high response rate only a slight bias in age, gender, education, and province was observed compared to standard Flemish demographics. At the Flemish level reported results were corrected for this bias. For the purpose of this paper—which is selecting new exposure indicators—representativity of the sample for the Flemish population is of lesser importance and no correction was made.

The questions of relevance for the current investigation are (English version obtained via Google Translate, original in brackets):

Q1.1: How satisfied are you generally with the quality of life (safety, child friendliness, environment, …) in your neighborhood? *<Hoe tevreden bent u in het algemeen over de leefkwaliteit (veiligheid, kindvriendelijkheid, leefmilieu, …) in uw buurt?>* Five point answering scale: very satisfied, satisfied more, less satisfied, not satisfied, not at all satisfied.
Q1.2: If we only look at the quality of life in your neighborhood, would you recommend friends and acquaintances to come here to live? *<Als we enkel kijken naar de leefkwaliteit in uw buurt, zou u vrienden en kennissen aanraden om hier te komen wonen?>* Two open answers: Why? Why not?
Q1.3: If you think about the past 12 months, to what extent are you annoyed or not annoyed by noise, odor or light in and around your home? *<Als u denkt aan de voorbije 12 maanden, in welke mate bent u gehinderd of niet gehinderd door geluid, geur of licht in en om uw woning?>* Five point answering categories for noise, odor, and light separately labeled: not at all, a little, moderately, highly, extremely.
Q1.8: Do you live in an environment with … five answering categories: very heavy traffic, heavy traffic, normal traffic, little traffic, very little traffic? <*Woont u in een omgeving met ... five answering categories: zeer veel verkeer, veel verkeer, normal verkeer, weinig verkeer, zeer weinig verkeer?*>
Q2.1: If you think about the past 12 months, how annoyed or not annoyed are you by the noise from the following sources in and around your home? *<Als u denkt aan de voorbije 12 maanden, hoe gehinderd of niet gehinderd bent u door het geluid van de volgende bronnen in en om uw woning?>* Five point answering categories for each source separately, labeled: not at all, a little, moderately, highly, extremely.
  Q2.1a: street traffic
  Q2.1b: rail traffic
  Q2.1c: air traffic

The annoyance questions closely follow the ISO/TS 15666:2003 standard [11].

### 2.2. Estimating Road Traffic Noise

This work surpasses prior work in the accuracy in estimating road traffic and road traffic noise exposure. For the population of Gent, a small city of roughly 350,000 inhabitants, the daily trip behavior of inhabitants is modeled in detail. This allows us on the one hand to estimate exposure to noise—and pollutants—while on the road, and at the other hand it gives us the opportunity to obtain much more detailed traffic intensity data for the smaller roads which are generally not or poorly included in city wide traffic models.

The model proposed for obtaining all trips starts from several sets of input data: the location of dwellings (postal addresses); the location of frequent trip destinations (e.g., shops, schools, employment) and their weights; the statistical database on travel habits of Flemish people. The latter database contains individual trip information about the daily number of trips, their purpose (hence destination), travel mode and the typical distance.

A first step in the modeling consists in generating all potential trips between dwellings and potential destinations (origin-destination combinations) for all modes separately. For car trips, the fastest route is selected, while for walking and biking the shortest route is used. To reduce computational burden, dwellings are grouped per street segment and the origin of trips is assumed at the center of the street. Once all potential trips are thus constructed, for each of the dwellings in the model area a household is randomly selected from the database on travel habits. The travel habits of this particular Flemish family are used to simulate the trip pattern for this dwelling, including the travel modes and routes. Thus for example families with children will be assigned school trips to a randomly chosen school at a distance corresponding to the typical travel time. This information determines the trip completely.

The trips are used in different ways in the model. Firstly, aggregating all trips per road section constitutes the local traffic in a road. This is added to the through traffic from a regional traffic model to obtain more accurate estimates of traffic intensities for smaller urban streets. Secondly, the trips are used to calculate exposure to air pollution and noise during travel for the members of every family included in the sample. For exposure during trips, a minimal distance of 10 m to the source is assumed even if both the receiver and the traffic follow the same route. When using motorized vehicles neither the emission of the vehicle itself nor the sound insulation of the vehicle are considered. Thirdly the trips determine the travel time to all essential destinations and thus give an indication of accessibility of these destinations.

**Figure 2.**
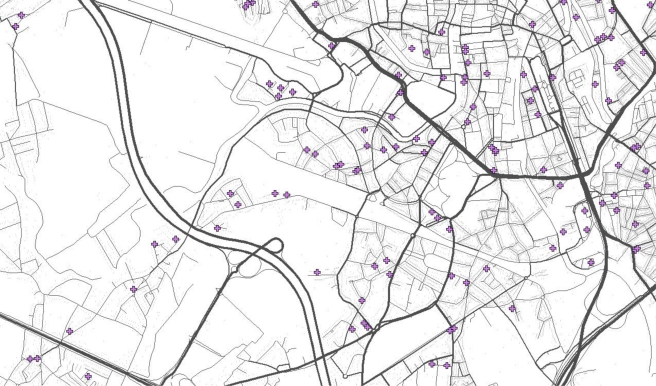
Map of part of the city of Gent showing the intensity of trips with local origin or destination as line thickness on a background of grey dots of dwelling addresses; crosses are survey point used in Section 3.

Figure 2 shows, as an example, part of the city of Gent with the thickness of streets corresponding to the intensity of trips made by inhabitants of the region travelling on these streets. This map is based on a sample of 10% of the 350,000 inhabitants of the study area. Three hundred thousand (300,000) trips made by this synthetic population were selected from 24 × 10^6^ potential routes that were generated in the first step.

Noise maps are constructed on the basis of the improved traffic estimates using the Harmonoise/Imagine [12] source model and ISO9613-2 propagation model taking into account the location and height of all buildings, but limiting the calculation to a single reflection and diffraction to improve calculation speed. It has been shown that in particular in urban areas, these approximations may underestimate both street canyon noise levels and shielded backyard levels [13]. To reduce computational cost, short term temporal fluctuations in noise levels are ignored reducing the calculation to hourly averaged L_Aeq_. In previous work we stressed the importance of notice events for explaining variations in reported noise annoyance [14] and illustrated the effect of temporal structure on perceived quality of open space [15,16]. By including traffic intensity in the models that were constructed, the effect of temporal structure can to some extent be reconstructed a posteriori. Finally, the potential positive influence of masking—both energetically and perceptually—by for example natural sounds, human vocalizations or the sound produced by the traveler itself has not been taken into account [17].

The proposed model allows quantifying exposure while moving from one location to another. This is potentially very useful for calculating health impact of traffic related air pollution [18]. Current air pollution maps have rather coarse calculation grids. This might be sufficient for some pollutants such as PM_10_ but others such as traffic related UFP (ultrafine particles) and NOx show much stronger spatial variations. On annual scale, traffic noise levels might be a good proxy for estimating the concentration of these pollutants—and their health impact—but on a day by day basis, meteorological effects introduce significant differences [19]. It should also be kept in mind that air pollution may not have a significant direct influence on reported quality of the living environment since persons may use proxies (like traffic intensity and traffic noise levels) anyhow while trying to judge the traffic related pollution level in their area.

### 2.3. Suitable Exposure Indicators

The enormous amount of exposure data generated by the model described in the previous section needs to be summarized in a small set of indicators for further analysis. For noise exposure at home, the average noise level during daytime, evening, and night-time L_den_ at the most exposed façade is included because of its importance in European noise mapping. Since local traffic is however only obtained during the day, we will limit the indicator to daytime only in most of the analyses. Since it was shown [20] that the availability of a quiet side can reduce reported noise annoyance, the level at the least exposed façade is also included in the analysis.

For noise exposure on the road, noise maps are sampled every 25 m. For one particular trip, the noise exposure needs to be spatially averaged over the route followed. Several alternatives were investigated: equivalent level over space, 10% highest levels, median level over space, or a total exposure indicator SEL = L_eq_ + 10 log(distance). The part of the trip included in the average was also varied. The first 300 m of the trip normally takes a person just outside its own street with the typical street geometry of the city of Gent. Alternatively the average over the whole trip, until the destination, was also considered, although it is expected that this indicator will incorporate effects which are too far from home to be considered part of the neighborhood.

When considering multiple trips, exposure also has to be aggregated over the different trips. For this the equivalent level, the median level, and the mathematical average of levels are considered. Because it is obvious that exposure to environmental noise will be masked by a person’s own vehicle when motorized transport is used, an aggregation only taking into account trips on foot or on bike is also included in the pool of indicators. Table 1 summarizes the indicators for noise exposure during trips that are considered in the analysis.

## 3. Results and Discussion

### 3.1. Analyses of the Survey Data

**Figure 3.**
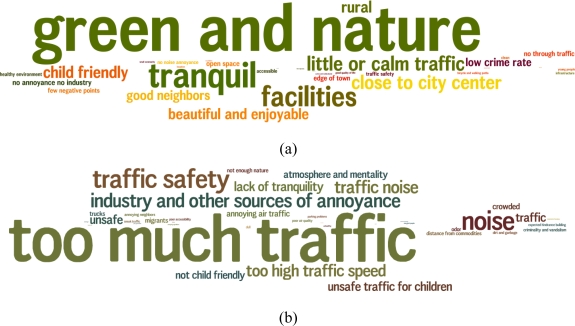
**(a)** Tag cloud of terms mentioned while asked why the respondent would suggest friend and acquaintances to come and live in his neighborhood; **(b)** same when asked why not; the size of the word corresponds to the frequency of occurrence, colors are used only to facilitate reading.

It is interesting to investigate first the response to the open question Q1.2 since it justifies the choice of variables included in the further analyses and modeling of exposure. In Figure 3 the words—or synonyms—that are frequently used by the survey respondents when mentioning reasons to recommend to friends and acquaintances to either come to (a) live in this neighborhood or (b) not, are shown. On the positive side the availability of green spaces and nature dominates, followed by a group of factors relating to tranquility and absence of traffic. Many respondents nevertheless also mention accessibility to city center and facilities as being important. The importance of green spaces and nature was previously investigated in detail by Gidlof-Gunnarsson *et al.* [21]. On the negative side too much traffic pops up together with several traffic-associated burdens such as traffic safety, unsafe traffic for children, too high traffic speed, traffic noise. In addition, noise in general and industry and other sources of annoyance are mentioned frequently as well as a lack of tranquility. Traffic and traffic noise thus seem of sufficient importance to merit a specific investigation, both in the positive as in the negative sense. This finding corresponds to the work by Leslie *et al.* [22] that identified traffic and traffic noise as one of the five principle component in a 17 item investigation of neighborhood satisfaction and showed a significant relationship with mental health. O.Campo *et al.* [23] based on concept mapping session also discussed traffic and noise, but with a much less prominent role. The latter study was done in Toronto, which is a completely different context which might explain these differences. It should however also be mentioned for completeness that the survey used in the underlying work was conducted by the regional government which might have urged participants to focus more on issues that are related to this level of governance and not on local urban issues.

To quantify these first observations and to identify potential pathways before proceeding with relating the survey to exposure indicators, relationships between answers on the survey questions are investigated. Since no exposure calculation is needed for this, the full statistical power of the region wide survey can be used.

**Figure 4.**
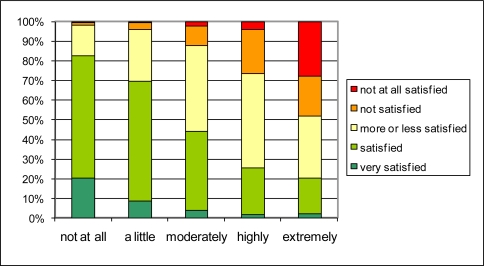
The influence of reported street traffic noise annoyance (Q2.1a) (horizontal) on global living quality in the neighborhood (Q1.1) (vertical).

**Figure 5.**
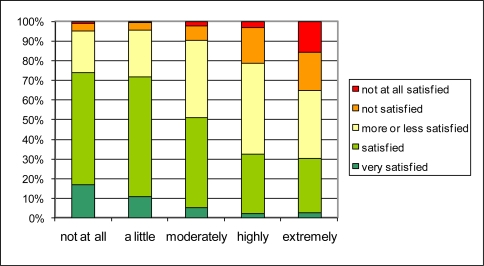
The influence of reported air traffic noise annoyance (Q2.1c) (horizontal) on global living quality in the neighborhood (Q1.1) (vertical).

The importance of traffic noise for the quality of life in the neighborhood that boiled up in the open question, is confirmed by plotting the answer to the question on quality of life in the neighborhood (Q1.1) against the answer to the question on noise annoyance at home caused by street traffic (Q2.1a) in Figure 4 and that caused by air traffic (Q2.1c) in Figure 5. People reporting to be highly or extremely annoyed by street traffic noise, are more likely to be not (or not at all) satisfied about the over-all living quality (resp. up to 30% and 50% of the people reporting highly or extremely annoyance). Only 20 to 25% of the people reporting high to extreme annoyance by street traffic noise, are still satisfied about their living quality. Reporting no annoyance by street traffic noise at all, seems to be sufficient for the large majority of people to report being satisfied to very satisfied with the overall quality of life in the neighborhood. Although the prevalence of being highly to extremely annoyed by air traffic noise is much lower (2.6%) than the prevalence of being highly to extremely annoyed by road traffic noise (11.5%), the effect on reported overall quality of the living environment is rather similar. In the categories of highly and extremely annoyed people, resp. over 20% and over 30% are not or not at all satisfied about the living quality. On the other hand, about 30% of these respondents are nevertheless satisfied or very satisfied about the living quality. It was also observed that this general trend is conserved when the population under study is limited to those living in a city or those living in smaller villages (not shown in the graphs). Both the insensitivity to the cause of noise annoyance and the insensitivity to living in a city or in a village on the countryside suggest that there is indeed a strong relationship between reported noise annoyance and reported quality of life in the neighborhood. The spread in the answers do not exclude—even suggest—the existence of other hidden spatially determined variables steering both noise annoyance and quality of life. Personal factors influencing sensitivity to the environment or reporting style can however not be ruled out at this point.

**Figure 6.**
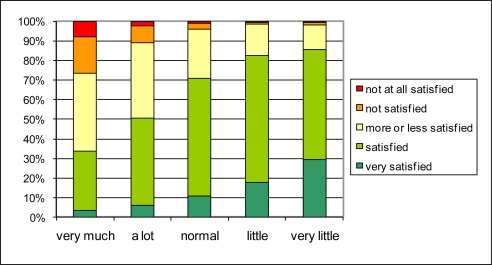
The effect of reported traffic intensity in the neighborhood (Q1.8) (horizontal) on global living quality in the neighborhood (Q1.1) (vertical).

The reported traffic intensity in the neighborhood (Q1.8) is related to satisfaction with the general quality of life in the neighborhood in a similar way as noise annoyance (Figure 6), but in general the relationship is less strong. From the people who judge that there is ‘very much traffic’ in their neighborhood, less than 30% is not or not all satisfied about the general living quality. In the category reporting ‘a lot of traffic’ in their neighborhood, about 10% is not (or not all) satisfied about the living quality. Reporting “very little traffic” results in 30% of the people reporting also being very satisfied with the quality of the living environment. At this end of the scale, reported traffic intensity is thus a somewhat stronger predictor for quality of life in the neighborhood than the absence of traffic noise annoyance.

**Figure 7.**
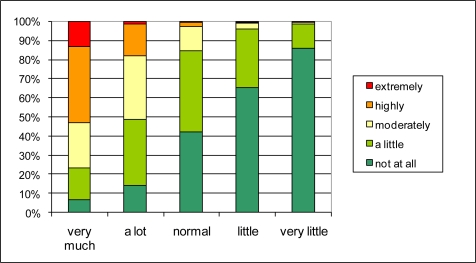
The effect of reported traffic intensity in the neighborhood (Q1.8) (horizontal) on the reported annoyance by street traffic noise (Q2.1a) (vertical).

**Figure 8.**
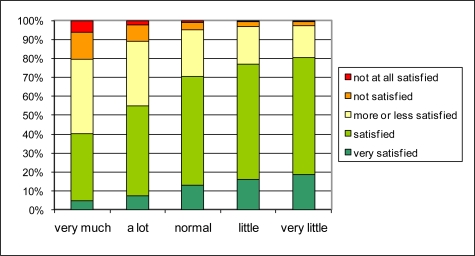
Probabilistic estimate of global living quality in the neighborhood (vertical) for different traffic intensities (horizontal); to be compared with Figure 6.

The connection between traffic intensity and quality of life in the neighborhood could exist through street traffic noise annoyance or through other negative or positive aspects of traffic such as safety, exhaust smell, or even accessibility. The distribution of answers on the street traffic noise annoyance question (Q2.1a) for different reported traffic intensities (Q1.8) in Figure 7 reveals a rather strong relationship. Over 50% of the people that report “very much traffic” also report high to extreme street traffic noise annoyance. Of those reporting little or very little traffic, no one reports high to extreme noise annoyance. The relationship between reported traffic intensity and quality of life in the neighborhood through street traffic noise annoyance seems possible. To investigate its probability further, the percentages in Figure 4 and Figure 7 are interpreted as conditional probabilities P(S_i_|A_j_) and P(A_j_|T_k_) respectively. S_i_ refers to the i^th^ degree of satisfaction with quality of life in the neighborhood; A_j_ refers to the j^th^ level of street traffic noise annoyance; and T_k_ to the k^th^ level of reported traffic intensity. P(S_i_|T_k_) can now be calculated in a probabilistic way as P(S_i_|T_k_) = ∑_j_ P(S_i_|A_j_) P(A_j_|T_k_). The result is shown in Figure 8. By comparing these results to Figure 6, it can be observed that the probabilistic approach slightly underestimates the percentage “not satisfied” to “not at all satisfied” in case of very much traffic and underestimates the percentage “very satisfied” in case of very little traffic, but that overall the agreement is strong. Thus the pathway through noise annoyance—that was the basic assumption for the probabilistic calculation—seems to be quite valid. The above mentioned deviations could be explained by other effects of traffic that impact the overall quality of life in both in positive (little or calm traffic in itself or child friendliness) and negative sense (traffic safety).

These conclusions form the basis for the analysis in the next paragraph, where several models are compared to explain and predict the perceived quality of life in a neighborhood. In this further analysis exposure indicators will form the main topic of interest.

### 3.2. Logistic Regression Model for Gent Data

The main outcome variable in this study is the reported general satisfaction with the quality of life in the neighborhood (Q1.1). This question is answered using a five point bipolar scale: very satisfied, satisfied, more or less satisfied, not satisfied, not at all satisfied. This coarse granulation can hardly be approximated as a continuous answer variable. In addition, it was shown that reasons mentioned in the open question differ significantly depending on whether they relate to positive or negative evaluation. For these reasons, the outcome is quantified either as ‘satisfied and very satisfied’ at the one hand and as ‘not satisfied and not at all satisfied’ at the other hand. A logistic regression model (without interaction terms) is used to quantify the importance of indicators studied: 1/(1 + exp[–z]), with z = β0 + β1 × 1 + β2 × 2 …

Since all indicators for exposure to noise during a trip (Table 1) are expected to be correlated (correlation coefficient reaches 0.7 in some cases), it is useful to first determine which ones are more appropriate to add to an overall logistic regression model. Therefore we first consider traffic noise annoyance in and around the house (Q2.1a). Answer categories are grouped to {moderately, highly, extremely} represented as 1 and {not at all, slightly} represented as 0 to balance the number of responses in each class. In Table 2 the p value in a chi square for each of the indicators for exposure during a trip is given when this indicator is added as an additional factor in a model based on the maximum façade exposure during the day, L_day,façade_. The latter indicator is always included in the model since it is most often used for exposure at home. L_day,facade_ has very high significance in the model with a p value of 1.7 × 10^−7^. The table shows that exposure within 300 m from the house taking an equivalent level over the length of each trip has the most significant effect. The method for averaging over different trips does not significantly affect this result. It should come as no surprise that an exposure level calculation over the first 300 m of a trip has exactly the same significance since most trips are over 300 m long. When trips are restricted to biking and walking, where exposure to external noise is expected to be more important, the influence of the parameter is largely reduced. This is mainly due to the introduction of uncertainty about the biking and walking habits of the surveyed persons. .

It comes as no surprise that the first 300 m of a trip are important for noise annoyance since the noise annoyance question explicitly refers to “in and around your house”. Therefore the same exercise is repeated for the question on general satisfaction with the quality of life in the neighborhood (Q1.1). Table 3 shows the p value for adding different indicators in a logistic model to the primary indicator L_day,façade_ for predicting the answers “satisfied” and “very satisfied” while Table 4 shows the same results for predicting the answers “not satisfied” to “not at all satisfied”. Grouping positive and negative evaluation respectively assures again a balanced number of responses in each class. Before interpreting these results it should be noted that the p value for façade exposure itself is much lower than in the case of the question on annoyance (0.013 and 0.0052 respectively). For the response category “not satisfied” to “not at all satisfied”, an equivalent level outperforms other methods for aggregating over different trips. The equivalent level is the aggregator that puts most weight in the most exposed trips. Just as for traffic noise annoyance an equivalent level over the first 300 m of a trip has a significant predictive effect. However, in the case of predicting “satisfaction” or “high satisfaction” with the quality of life of a neighborhood, also the exposure level during whole trips on foot or by bike pop up as highly significant.

From the above analyses it is concluded that $L_{T,300,\text{eq}}^{\text{eq}}$ is the first candidate as an indicator for exposure to noise during trips. A second candidate, especially when satisfaction with the general quality of the living environment is at stake, could be $L_{\text{BP},w,\text{sel}}^{\text{eq}}$. Strictly speaking, a linear average over dB values for different trips has a slightly more significant effect, but for the sake of simplicity, it was decided to stick to the same aggregation procedure for both trip-related indicators.

**Figure 9.**
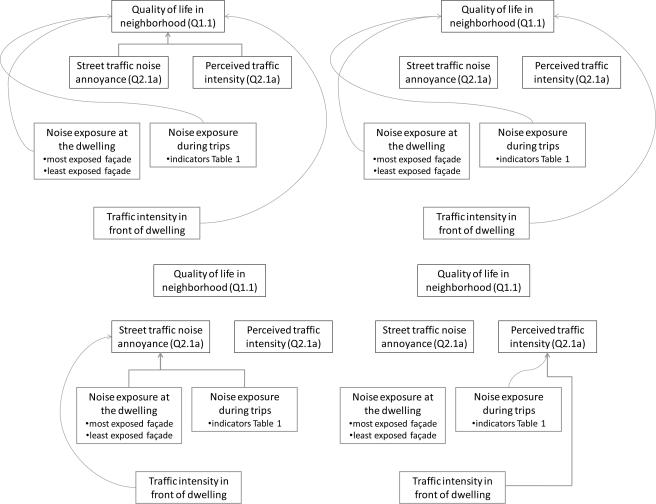
Models studied using logistic regression: a model for QoL including questionnaire response and data from GIS (upper left); a model for QoL including only GIS data (upper right); sub models for street traffic noise annoyance and perceived traffic intensity (lower left and right).

With the knowledge on suitable indicators for noise exposure during a trip in mind, a multiple logistic regression model for the influence of traffic noise on satisfaction with the quality of life in the neighborhood (Figure 9) is now constructed.

When constructing multiple regression models with parameters that are not orthogonal, one could either look for principle components first or account for the order in which parameters are entered. Exposure indicators used in this study are mutually dependent but have physical relevance clearly linked to neighborhood situations and some are commonly used in environmental noise assessment. Therefore it is preferred not to combine them in a principle component. Moreover this approach will allow finding the best indicators to add to common practice. This choice implies that all parameters will need to be entered in the models in different orders. The first model contains variables taken from the survey (Q2.1a on traffic noise annoyance and Q1.8 on traffic load of the neighborhood) but also the noise exposure variables (*L_day,façade_*, *L_day,quiet_*, the traffic noise level at the quiet side of the house, $L_{T,300,\text{eq}}^{\text{eq}}$, $L_{\text{BP},w,\text{sel}}^{\text{eq}}$) selected above and the traffic intensity on the road in front of the house (*N_street_*). In the full model, the variables are added in different orders, in order to evaluate the (added) significance of each specific variable set. The variables taken from the survey have the highest significance even when added to the model last (Table 5). Survey results are indeed expected to be the best estimate of subjective evaluation of noise exposure since they account for personal factors that might influence the way the sonic environment is perceived or the way a person reports about it [24]. In addition, using reported annoyance and traffic intensity avoids potential errors in the noise exposure model or the model accounting for the behavior of the respondents. It is interesting to investigate whether exposure to traffic noise—as measured by the proposed indicators—has an influence on reported satisfaction with the quality of life in the neighborhood that is not captured by the question on noise annoyance at home and the question on perceived traffic intensity in the neighborhood. Therefore the performance of a model including only these questionnaire variables is compared to the full model. The ANOVA test results in Table 6 show that there is a moderately significant improvement by adding exposure indicators for predicting satisfaction with the quality of life in the neighborhood but not for predicting “not satisfied” to “not at all satisfied”. The availability of a quiet side, measured by *L_day,quiet_*, and quiet walking and biking routes near the house, measured by $L_{\text{BP},w,\text{sel}}^{\text{eq}}$, could be a proxy for the availability of tranquility and availability of green and nature, that were mentioned in the open question as a positive factor, but less prominent as negative factors.

For many practical applications, a model for quality of the living environment should not depend on questions in a survey. Hence, the response to question Q1.8 on traffic intensity and Q2.1a on road traffic noise annoyance, were removed from the model. The significance of the different factors remains the same as can be seen from Table 5. However, when $L_{T,300,\text{eq}}^{\text{eq}}$ is entered in the model first (model 2b), it becomes the most significant factor, performing even better than *L_day,façade_* in model 2a. Adding L_day,façade_ no longer improves the model, as can be seen from the chi squared test on the comparison between models in Table 6. Adding an additional indicator for exposure during trips that is specifically targeting trips made on foot or by bike, $L_{\text{BP},w,\text{sel}}^{\text{eq}}$, has a significant influence in the model for predicting satisfaction with the quality of life in the neighborhood, but not for predicting dissatisfaction. A similar conclusion hold for adding the quiet side indicator, *L_day,quiet_*, but adding both factors does not add any new value to the model. The fact that only satisfaction is predicted more accurately shows that a quiet side and quiet (and green) biking or walking routes are not missed when absent and their absence is not a negative aspect for the neighborhood, but that their presence is perceived as an asset for the living quality. This is again confirmed by the open question results shown in Figure 3.

Still, the model based on exposure only, performs significantly worse than the model including noise annoyance and traffic intensity questions from the survey (Table 6). Thus it is useful to investigate whether traffic noise annoyance and reported (subjective) traffic intensity can accurately be modeled based on exposure indicators (the lower models in Figure 9). In Table 7 the significance of several exposure indicators in a logistic model for predicting street traffic noise annoyance and reported traffic intensities are shown. The indicators that come out significant in the noise annoyance model differ depending on the level of annoyance. For high to extreme annoyance, the noise level at the most exposed façade is the only significant indicator while for moderate to extreme annoyance and for “no annoyance at all”, the exposure during trips should be added as an indicator. Previous work showed the importance of the noise level at the quiet side for perceived noise annoyance [20] but this effect could not be found here, probably because the noise level at the least exposed façade was not determined accurately enough.

Table 8 shows that a model for moderate to extreme annoyance and a model for the absence of annoyance improve statistically significantly by adding the indicator for exposure during the first 300 m of trips: $L_{T,300,\text{eq}}^{\text{eq}}$. Adding additional factors to the model gives no significant improvement. In contrast to the model for satisfaction with the quality of life in the neighborhood, *L_day,façade_* remains an important indicator. The geographical area referred to in the noise annoyance question: in and around your house, focuses attention more on the façade than the quality of life question that refers to the neighborhood, which could explain this difference. It was nevertheless shown in previous work by Klaboe *et al.* [25] that the soundscape in the wider area also matters for rating noise annoyance at home, which seems to be confirmed here, at least if moderate to extreme annoyance is considered.

In the multiple logistic models for reported traffic intensity (Table 7), the number of vehicles in the street in front of the house, N_street_, comes out rather insignificant when noise exposure indicators are added first. When N_street_ is added first, it becomes strongly significant for predicting heavy and very heavy traffic but not for predicting little traffic. In both cases noise exposure during trips is very significant. The model comparison in Table 9 confirms that adding noise exposure during the first part of trips helps very significantly in predicting both reported intensities of traffic. Thus, ${\text{L}}_{\text{T},300,\text{eq}}^{\text{eq}}$—although initially designed for predicting the quality of life in a neighborhood—also mimics very well how persons sample traffic intensity in their neighborhood. The most obvious explanation is that *N_street_* only incorporates the traffic in the own street, while the noise indicator adds up the traffic in a larger area around the house. Another explanation might include the way people perceive traffic intensity which might include the noise level.

To conclude and summarize, the β coefficients in the multiple linear regression models that include the minimal number of factors are given in Table 10.

## 4. Conclusions

The relationship between traffic noise and perceived quality of life in the neighborhood was investigated by comparing the results of a survey with new types of exposure indicators focusing on noise exposure during trips from the house. The latter are calculated in an innovative way by sampling origins, destinations (shops, schools, *etc.*) and typical travel behavior from several databases and reconstructing all possible trips leaving the dwelling.

The importance of traffic and traffic noise in reported quality of life in a neighborhood is observed by analyzing open questions on positive and negative aspects of coming to live in this neighborhood; by analyzing the relationship between the quality of life question and a standard noise annoyance question, and most importantly, by obtaining multiple logistic models relating quality of life in the neighborhood to noise exposure. The relationship between reported noise annoyance and quality of the living environment suggest that the pathway is a direct one, not relying on underlying hidden variables. Combining this analysis with a question on traffic intensity in the neighborhood further suggests that the pathway from traffic through noise to quality of life in the neighborhood accounts for the strongest relationship between traffic and quality of life. Other paths may contribute, but most probably to a much lesser extent.

Traffic noise exposure in the neighborhood is assessed by estimating where people would drive their car and where they would walk close to their house while leaving for work, school, shopping or whatever other reason. In that way, the exposure indicators for noise exposure during trips account for the access routes and the location of the most important attraction poles close to the house, rather than merely considering a circular area around the house as the neighborhood. Several ways of aggregating noise over the length of the trip and between trips are compared. Statistical analysis showed that calculating an equivalent level over the first 300 m of each trip and aggregating over all trips using an equivalent level as well, $L_{T,300,\text{eq}}^{\text{eq}}$, results in the most significant improvement of models for noise annoyance at home and quality of the living environment. In addition, a restriction to trips made on foot or by bike improves the predictability of satisfaction with the quality of the living environment, but not of dissatisfaction with it. For noise annoyance at home, the level at the most exposed façade is still a dominant indicator. Adding the above mentioned indicator for noise exposure during trips improves the model for moderate to extreme annoyance and also the model for no annoyance at all. The positive effect of access to a quiet side on noise annoyance at home is not recovered probably because quiet side levels were not calculated accurately enough.

Most surprisingly at first sight, the indicator for noise exposure during trips is the most significant contributor to a model for quality of life in the neighborhood. Adding façade exposure to the model gives no improvement, which is a rather surprising result that is however understandable since the neighborhood has a wider spatial meaning than just one’s own dwelling. Similarly surprising is the observation that the same exposure indicator performs best in a model for perceived traffic intensity in the neighborhood, more so than a traffic count on the street of the dwelling itself. At this point, it is only possible to propose a few hypotheses to explain this observation: traffic intensity might be judges via noise or the traffic aggregation embedded in the noise exposure indicator might be just the way to aggregate traffic intensities over an area that corresponds best to perceptive evaluation.

The logistic models obtained in this work provide an interesting step forward for building a general model for evaluating the overall impact of land use planning and mobility planning on the quality of life of a neighborhood.

## Figures and Tables

**Table 1.** Indicators for noise exposure during trips.

			Exposure aggregation over a single trip							
			Equivalent level		10% highest		Median		Exposure	
			300 m	whole trip	300 m	whole trip	300 m	whole trip	300 m	whole trip
Agg. over different trips	All modes	Equivalent	$L_{T,300,\text{eq}}^{\text{eq}}$	$L_{T,w,\text{eq}}^{\text{eq}}$	$L_{T,300,10}^{\text{eq}}$	$L_{T,w,10}^{\text{eq}}$	$L_{T,300,50}^{\text{eq}}$	$L_{T,w,50}^{\text{eq}}$	$L_{T,300,\text{sel}}^{\text{eq}}$	$L_{T,w,\text{sel}}^{\text{eq}}$
		Median	$L_{T,300,\text{eq}}^{50}$	$L_{T,w,\text{eq}}^{50}$	$L_{T,300,10}^{50}$	$L_{T,w,10}^{50}$	$L_{T,300,50}^{50}$	$L_{T,w,50}^{50}$	$L_{T,300,\text{sel}}^{50}$	$L_{T,w,\text{dB}}^{50}$
		Linear average of dB values	$L_{T,300,\text{eq}}^{\text{dB}}$	$L_{T,w,\text{eq}}^{\text{dB}}$	$L_{T,300,10}^{\text{dB}}$	$L_{T,w,10}^{\text{dB}}$	$L_{T,300,50}^{\text{dB}}$	$L_{T,w,50}^{\text{dB}}$	$L_{T,300,\text{sel}}^{\text{dB}}$	$L_{T,w,\text{sel}}^{\text{dB}}$
	Bike and pedestrian	Equivalent	$L_{\text{BP},300,\text{eq}}^{\text{eq}}$	$L_{\text{BP},w,\text{eq}}^{\text{eq}}$	$L_{\text{BP},300,10}^{\text{eq}}$	$L_{\text{BP},w,10}^{\text{eq}}$	$L_{\text{BP},300,50}^{\text{eq}}$	$L_{\text{BP},w,50}^{\text{eq}}$	$L_{\text{BP},300,\text{sel}}^{\text{eq}}$	$L_{\text{BP},w,\text{sel}}^{\text{eq}}$
		Median	$L_{\text{BP},300,\text{eq}}^{50}$	$L_{T,w,\text{eq}}^{50}$	$L_{T,300,10}^{50}$	$L_{T,w,10}^{50}$	$L_{T,300,50}^{50}$	$L_{T,w,50}^{50}$	$L_{T,300,\text{sel}}^{50}$	$L_{T,w,\text{sel}}^{50}$
		Linear average of dB values	$L_{\text{BP},300,\text{eq}}^{\text{dB}}$	$L_{\text{BP},w,\text{eq}}^{\text{dB}}$	$L_{\text{BP},300,10}^{\text{dB}}$	$L_{\text{BP},w,10}^{\text{dB}}$	$L_{\text{BP},300,50}^{\text{dB}}$	$L_{\text{BP},w,50}^{\text{dB}}$	$L_{\text{BP},300,\text{sel}}^{\text{dB}}$	$L_{\text{BP},w,\text{sel}}^{\text{dB}}$

**Table 2.** p in chi square test for a logistic model predicting moderate, high or extreme traffic noise annoyance (Q2.1a) on the basis on L_day_ on the most exposed façade and the additional indicator in the table. The value of p for L_day,facade_ is 1.7 × 10^−7^.

			Exposure aggregation over a single trip							
			Equivalent level		10% highest		Median		Exposure	
			300 m	whole trip	300 m	whole trip	300 m	whole trip	300m	whole trip
Agg. over different trips	All modes	Equivalent	0.0016 **	0.09		0.02 *	0.01 *	0.12	0.0016 **	0.22
		Median	0.0015 **	0.12		0.05 *	0.01 *	0.15	0.0015 **	0.25
		Linear average of dB values	0.0016 **	0.10		0.04*	0.01 *	0.13	0.0016 **	0.22
	Bike and pedestrian	Equivalent	0.02 *	0.20		0.78	0.11	0.19	0.02 *	0.36
		Median	0.02 *	0.27		0.87	0.15	0.28	0.02 *	0.5
		Linear average of dB values	0.02 *	0.24		0.92	0.14	0.34	0.02*	0.41

* p < 0.05,

** p < 0.01 and

*** p < 0.001.

**Table 3.** p in chi square test for a logistic model predicting satisfied to very satisfied with general quality of life of the neighborhood (Q1.1) on the basis on L_day_ on the most exposed façade and the additional indicator in the table. The value of p for L_day,facade_ is 0.013.

			Exposure aggregation over a single trip							
			Equivalent level		10% highest		Median		Exposure	
			300 m	whole trip	300 m	whole trip	300 m	whole trip	300 m	whole trip
Agg. over different trips	All modes	Equivalent	0.0086 **	0.081		0.81	0.16	0.19	0.0086 **	0.018 *
		Median	0.021 *	0.055		0.91	0.23	0.13	0.021 *	0.012 *
		Linear average of dB values	0.031 *	0.045*			0.32	0.12	0.032 *	0.0081 **
	Bike and pedestrian	Equivalent	0.094	0.095		0.69	0.47	0.87	0.095	0.0034 **
		Median	0.18	0.091		0.41	0.73	0.93	0.18	0.0031 **
		Linear average of dB values	0.22	0.032 *			0.86	0.64	0.22	0.0012 **

* p < 0.05,

** p < 0.01 and

*** p < 0.001.

**Table 4.** p in chi square test for a logistic model predicting not satisfied to not at all satisfied with general quality of life of the neighborhood (Q1.1) on the basis on L_day_ on the most exposed façade and the additional indicator in the table. The value of p for L_day,facade_ is 0.0053.

			Exposure aggregation over a single trip							
			Equivalent level		10% highest		Median		Exposure	
			300 m	whole trip	300 m	whole trip	300 m	whole trip	300 m	whole trip
Agg. over different trips	All modes	Equivalent	0.036 *	0.49		0.096	0.15	0.23	0.036 *	0.98
		Median	0.084	0.57		0.25	0.22	0.33	0.084	0.97
		Linear average of dB values	0.10	0.67			0.26	0.37	0.10	0.81
	Bike and pedestrian	Equivalent	0.33	0.21		0.31	0.53	0.06	0.33	0.85
		Median	0.54	0.28		0.60	0.73	0.12	0.54	0.94
		Linear average of dB values	0.54	0.37			0.74	0.13	0.54	0.95

* p < 0.05,

** p < 0.01 and

*** p < 0.001.

**Table 5.** p in chi squared test for logistic model predicting satisfaction with the quality of life in the neighborhood (Q1.1); label “not satisfied” covers the last two response categories on the five point scale, the label “satisfied” the first two response categories.

		***L****_day,façade_*	***L****_day,quiet_*	***N****_street_*	${\text{L}}_{\text{T},300,\text{eq}}^{\text{eq}}$	${\text{L}}_{\text{BP},\text{w},\text{sel}}^{\text{eq}}$	**Q1.8**	**Q2.1a**
model 1a	order entered	1	2	3	4		5	6
	not satisfied	0.0053 **	0.47	0.14	0.061		0.0015 **	0.00060 ***
	satisfied	0.013 *	0.0056 **	0.44	0.010 *		3.0 × 10^−7^***	2.4 ×10^−8^***
model 1b	order entered	3	4	5	6	7	1	2
	not satisfied	0.52	0.34	0.38	0.36	0.92	9.1 × 10^−6^***	0.00060 ***
	satisfied	0.73	0.0019 **	1.00	0.56	0.018 *	8.6 ×10^−10^***	4.6 × 10^−8^***
model 2a	order entered	1	2	3	4	5		
	not satisfied	0.0053 **	0.47	0.14	0.061	0.75		
	satisfied	0.013 *	0.0056 **	0.44	0.010 *	0.041 *		
model 3	order entered	2	3	4	1			
	not satisfied	0.42	0.40	0.29	0.00069 ***			
	satisfied	0.73	0.0051 **	0.78	0.00033 ***			
model 2b	order entered	3	4	5	1	2		
	not satisfied	0.42	0.36	0.31	0.00069 ***	0.90		
	satisfied	0.53	0.053	0.71	0.00033 ***	0.0048 **		

* p < 0.05,

** p < 0.01 and

*** p < 0.001.

**Table 6.** p value in ANOVA chi squared test comparing different models for predicting satisfaction with quality of life in the neighborhood (Q1.1).

	Base model	Compare model	P
not satisfied	model 2a	model 1b	1.1 × 10^−5^***
	Q1.8 and Q2.1a	model 1b	0.70
	*L_day,façade_*	*L_day,façade_+*$L_{T,300,\text{eq}}^{\text{eq}}$	0.036 *
	$L_{T,300,\text{eq}}^{\text{eq}}$	$L_{T,300,\text{eq}}^{\text{eq}}$ + *L_day,façade_*	0.42
	$L_{T,300,\text{eq}}^{\text{eq}}$	$L_{T,300,\text{eq}}^{\text{eq}}$ + $L_{\text{BP},w,\text{sel}}^{\text{eq}}$	0.89
Satisfied	model 2a	model 1b	9.6 × 10^−14^***
	Q1.8 and Q2.1a	model 1b	0.008 **
	*L_day,façade_*	*L_day,façade_+*$L_{T,300,\text{eq}}^{\text{eq}}$	0.0086 **
	$L_{T,300,\text{eq}}^{\text{eq}}$	$L_{T,300,\text{eq}}^{\text{eq}}$ + *L_day,façade_*	0.73
	$L_{T,300,\text{eq}}^{\text{eq}}$	$L_{T,300,\text{eq}}^{\text{eq}}$ + $L_{\text{BP},w,\text{sel}}^{\text{eq}}$	0.0048 **

* p < 0.05,

** p < 0.01 and

*** p < 0.001.

**Table 7.** p in chi squared test for logistic model predicting street traffic noise annoyance and reported traffic intensities.

		***L****_day,façade_*	***L****_day,quiet_*	***N****_street_*	${\text{L}}_{\text{T},300,\text{eq}}^{\text{eq}}$
Street traffic noise annoyance (Q2.1a)	order entered	1	2	3	4
	high to extreme	0.0034 **	0.74	0.69	0.47
	moderate to extreme	1.7 × 10^−7^***	0.35	0.035*	0.0052 **
	not at all	2.4 × 10^−6^***	0.55	0.51	2.5 × 10^−5^***
Reported traffic intensity (Q1.8)	order entered	1	2	3	4
	little to very little	6.2 × 10^−5^***	0.046 *	0.56	2.7 × 10^−6^***
	heavy to very heavy	3.4 × 10^−10^***	0.96	0.0089 **	1.1 × 10^−8^***
Reported traffic intensity (Q1.8)	order entered	3	4	1	2
	little to very little	0.22	0.15	0.14	1.3 × 10^−9^***
	heavy to very heavy	0.36	0.21	1.4 10^−4^***	3.3 × 10^−15^***

* p < 0.05,

** p < 0.01 and

*** p < 0.001.

**Table 8.** p value in ANOVA chi squared test comparing different models for predicting reported street traffic noise annoyance (Q2.1a).

	**Base model**	**Compare model**	**p**
high to extreme	*L_day,façade_*	Full model of Table 7	0.85
moderate to extreme	*L_day,façade_*	*L_day,façade_+*$L_{T,300,\text{eq}}^{\text{eq}}$	0.0017 **
	*L_day,façade_+*$L_{T,300,\text{eq}}^{\text{eq}}$	Full model of Table 7	0.20
not at all	*L_day,façade_*	*L_day,façade_+*$L_{T,300,\text{eq}}^{\text{eq}}$	1.8 × 10^−5^***
	*L_day,façade_+*$L_{T,300,\text{eq}}^{\text{eq}}$	Full model of Table 7	0.92

* p < 0.05,

** p < 0.01 and

*** p < 0.001.

**Table 9.** p value in ANOVA chi squared test comparing different models for predicting reported traffic intensity in the neighborhood (Q1.8).

	**Base model**	**Compare model**	**P**
little to very little	*N*_street_	*N*_street_*+*$L_{T,300,\text{eq}}^{\text{eq}}$	1.3 × 10^−9^***
	*N*_street_*+*$L_{T,300,\text{eq}}^{\text{eq}}$	Full model of Table 7	0.18
heavy to very heavy	*N_street_*	*N*_street_*+*$L_{T,300,\text{eq}}^{\text{eq}}$	3.3 × 10^−15^***
	*N*_street_*+*$L_{T,300,\text{eq}}^{\text{eq}}$	Full model of Table 7	0.31

* p < 0.05,

** p < 0.01 and

*** p < 0.001.

**Table 10.** β coefficients in the multiple logistic regression models that were retained.

		***L****_day,façade_*	${\text{L}}_{\text{T},300,\text{eq}}^{\text{eq}}$	${\text{L}}_{\text{BP},\text{w},\text{sel}}^{\text{eq}}$	***N****_street_*	***Constant***
Street traffic noise annoyance (Q2.1a)	High to extreme	0.046				–4.14
	Moderate to extreme	0.034	0.051			–5.33
	Not at all	–0.023	–0.075			4.55
Reported traffic intensity (Q1.8)	Little to very little		–0.11		1.68 × 10^−6^	4.53
	Heavy to very heavy		0.11		4.19 × 10^−5^	–6.26
Satisfaction with quality of life in neighborhood (Q1.1)	Satisfied		–0.045	0.084		–4.78
	Not satisfied		0.057			–5.14
